# Integrated summary of immunogenicity of polatuzumab vedotin in patients with relapsed or refractory B-cell non-Hodgkin’s lymphoma

**DOI:** 10.3389/fimmu.2023.1119510

**Published:** 2023-03-29

**Authors:** Randall C. Dere, Richard L. Beardsley, Dan Lu, Tong Lu, Grace H-W. Ku, Gabriel Man, Van Nguyen, Surinder Kaur

**Affiliations:** ^1^ Department of BioAnalytical Sciences, Genentech, Inc., South, San Francisco, CA, United States; ^2^ Department of Analytical Development and Quality Control, Genentech, Inc., South San Francisco, CA, United States; ^3^ Department of Clinical Pharmacology Oncology, Genentech, Inc., South San Francisco, CA, United States; ^4^ Department of Product Development Hematology, Genentech, Inc., South San Francisco, CA, United States; ^5^ Department of Product Development Safety, Genentech, Inc., South San Francisco, CA, United States

**Keywords:** POLIVY ^®^, polatuzumab vedotin, antibody-drug conjugate, integrated summary of immunogenicity, diffuse large B- cell lymphoma

## Abstract

Polatuzumab vedotin, marketed under the trade name POLIVY^®^, is a CD79b-targeted antibody-drug conjugate that preferentially delivers a potent anti-mitotic agent (monomethyl auristatin E) to B cells, resulting in anti-cancer activity against B-cell malignancies. In 2019, polatuzumab vedotin in combination with rituximab and bendamustine was approved by the United States Food and Drug Administration for the treatment of adult patients with diffuse large B-cell lymphoma who have received at least two prior therapies. Recent Health Authority guidance recommendations for submitting an Integrated Summary of Immunogenicity were followed including a comprehensive immunogenicity risk assessment, bioanalytical strategy, and immunogenicity data to support the registration of polatuzumab vedotin. Key components of the polatuzumab vedotin Integrated Summary of Immunogenicity and data are presented. Validated semi-homogeneous bridging enzyme-linked immunosorbent assays were used to detect anti-drug antibodies (ADA) to polatuzumab vedotin and characterize the immune response in patients with non-Hodgkin’s lymphoma. The overall incidence of ADA observed for polatuzumab vedotin was low across seven clinical trials. The low incidence of ADA is likely due to the mechanism of action of polatuzumab vedotin that involves targeting and killing of B cells, thereby limiting the development to plasma cells and ADA secretion. Furthermore, patients are co-medicated with rituximab, which also targets B cells and results in B-cell depletion. Therefore, the immunogenicity risk is considered low and not expected to impact the polatuzumab vedotin benefit/risk profile.

## Introduction

Diffuse large B-cell lymphoma (DLBCL) is an aggressive form of Non-Hodgkin’s lymphoma (NHL), with a median survival of less than one year if left untreated. Approximately 60% of patients may be cured with rituximab, cyclophosphamide, doxorubicin, vincristine, and prednisone (R-CHOP), the current front-line standard of care (1). However, about one-third of patients will develop relapsed or refractory (R/R) disease, which remains a major cause of morbidity and mortality (2). If front-line therapy fails, the current standard second-line approaches for young and fit patients with R/R DLBCL include intensive chemotherapy regimens including R-ICE (rituximab, ifosfamide, carboplatin, etoposide), R-DHAP (rituximab, dexamethasone, cytarabine, cisplatin), or R-GDP (rituximab, gemcitabine, dexamethasone, cisplatin or carboplatin), followed by autologous stem cell transplant. However, as a result of toxicity, these approaches are not feasible options in those who are deemed “transplant ineligible” due to older age or comorbidities. With currently available second-line therapy options, the outcome of such patients is poor with generally no chance of prolonged periods of disease control (3). Polatuzumab vedotin in combination with rituximab and bendamustine was approved in the USA, EU, and other countries for the treatment of adult patients with DLBCL who have received prior therapies. Polatuzumab vedotin in combination with rituximab, cyclophosphamide, doxorubicin and prednisone was subsequently approved in the EU, Japan, and other countries for the treatment of patients with previously untreated DLBCL.

Polatuzumab vedotin is a CD79b-targeted antibody-drug conjugate (ADC) that preferentially delivers a potent anti-mitotic agent, monomethyl auristatin E (MMAE) (4), to B cells, which results in anti-cancer activity against B-cell malignancies (5). The polatuzumab vedotin molecule consists of MMAE covalently attached to a CD79b-directed humanized immunoglobulin (Ig) G1 monoclonal antibody through a protease-cleavable linker, maleimidocaproyl-valine-citrulline-*p*-aminobenzyloxycarbonyl (mc-vc-PAB) (5). The polatuzumab vedotin production process was designed to deliver an average of 3.5 linked MMAE moieties per antibody (6). Polatuzumab vedotin specifically binds human CD79b, a signaling component of the B-cell receptor located on the surface of B-cells (7). As such, CD79b expression is restricted to normal cells within the B-cell lineage (with the exception of plasma cells) and malignant B-cells and is expressed in >95% of DLBCLs (5, 8–13). Therefore, targeted delivery of MMAE is expected to be restricted to these cells. Upon binding to CD79b, polatuzumab vedotin is rapidly internalized and the linker is cleaved by lysosomal proteases, leading to intracellular release of MMAE (12, 14–16). The released MMAE subsequently binds to microtubules, causing inhibition of cell division and apoptosis induction, and ultimately cell death (17–19).

The polatuzumab vedotin clinical development included a comprehensive immunogenicity assessment and an Integrated Summary of Immunogenicity was provided to support the registration. The Integrated Summary of Immunogenicity included an immunogenicity risk assessment, bioanalytical strategy, and clinical immunogenicity assessment. The clinical assessment included anti-drug antibody (ADA) data obtained from seven clinical studies where polatuzumab vedotin was administered as a single agent or in combination with other agents to patients with relapsed or refractory B-cell NHL. In all clinical studies, polatuzumab vedotin was administered by intravenous infusion over 30 to 90 minutes. These studies have characterized single- and multiple-dose pharmacokinetics (PK) of three key analytes and the immunogenicity of polatuzumab vedotin (20–25), administered as either liquid drug product (used in Studies DCS4968g [NCT01290549], GO27834 [NCT01691898], GO29044 [NCT01992653], GO29365 [NCT02257567]) or lyophilized drug product (used in Studies GO29833 [NCT02611323], GO29834 [NCT02600897], BO29561 [NCT02729896]). The pivotal study GO29365 subsequently added two additional cohorts using lyophilized drug product. Data from these additional cohorts were not included in the Integrated Summary of Immunogenicity.

The purpose of this article is to provide a comprehensive immunogenicity risk assessment of polatuzumab vedotin for human use and to summarize the immunogenicity data included in the Integrated Summary of Immunogenicity that supported the registration of polatuzumab vedotin in combination with rituximab and bendamustine.

## Methods

### Sampling for immunogenicity testing

For pivotal study GO29365, serum samples for ADA assessment were collected at pre-infusion cycles 1, 2, and 4, at treatment completion or early termination, and at post-treatment visits (2, 4, 6, 9, and 12 months). Sample collection frequency was reduced from the earlier DCS4968g study (every cycle up to 8 cycles, at treatment completion or early termination, and at post-treatment visits) based on the median cycle to onset of ADA (cycle 3).

### Screening assay

The validated immunogenicity assays to detect polatuzumab vedotin ADAs utilized a semi-homogenous bridging enzyme-linked immunosorbent assay format employing biotin- and digoxigenin-ADC reagents, as previously described (26). Briefly, the conjugated reagents (2 µg/mL) were co-incubated overnight with human serum samples and controls diluted 1/50 in assay diluent (50 mM phosphate buffered saline/0.5% bovine serum albumin/0.05% polysorbate 20/0.05% Proclin 300, pH 7.4) to form immunocomplexes. The mixture was transferred to a streptavidin-coated 96-well plate (StreptaWell™ High Bind; Roche Diagnostics, Indianapolis, IN). After incubation and a wash step, horseradish peroxidase (HRP)-conjugated mouse anti-digoxin monoclonal antibody (mAb; Jackson ImmunoResearch Labs, PA) was added for detection. Tetramethyl benzidine (TMB, KPL Inc., MD) was added for color development, and the reaction was stopped by adding 1 M phosphoric acid. The plates were read on a plate reader at 450 nm for detection and 630 nm for reference absorbance. The ADA screening assay was optimized to tolerate drug interference and detected the positive control sample at 90 ng/mL in the presence of 20 μg/mL of polatuzumab vedotin, which is greater than the expected circulating concentrations at the time of sample collection.

### Confirmatory assay

The specificity of the immune response for samples that screened positive for ADA was confirmed by competitive binding with polatuzumab vedotin. The validated ADA confirmatory assay method was similar to the screening assay procedure except the appropriate controls and samples were pre-incubated with 30 µg/mL of polatuzumab vedotin prior to co-incubation with the conjugated reagents.

### Domain specificity assay

The exploratory domain specificity assay method was similar to the screening assay procedure except the appropriate controls and samples were pre-incubated with 30 µg/mL of unconjugated polatuzumab vedotin antibody prior to co-incubation with the conjugated reagents.

### Titer assay

The validated titration assay method was similar to the screening assay procedure except the appropriate controls and samples were diluted to minimum required dilution (MRD) of 1/50 and serially diluted two-fold prior to co-incubation with the conjugated reagents. Antibody titer values were determined using a log titer data reduction program. The minimal reportable titer of the assay was log_10_50 = 1.70 log titer units.

### Neutralizing antibody assay

The validated polatuzumab vedotin NAb assay utilized a cell-based format (Nguyen et al., manuscript in preparation). A sample pre-treatment procedure was required to minimize the polatuzumab vedotin interference in the assay. Specifically, polatuzumab vedotin-specific ADAs will be extracted from the samples using a modified BEAD (Bead Extraction with Acid Dissociation) method. A Burkitt lymphoma cell line (BJAB) was selected as the host cell based on its favorable reactivity to polatuzumab vedotin, and the desirable signal/noise ratio in the assay. A rabbit anti-polatuzumab vedotin polyclonal antibody enriched for anti-complementarity determining region (CDR) and anti-MMAE antibodies was chosen as the surrogate positive control. BJAB cells were incubated with polatuzumab vedotin in the presence of BEAD-processed ADA-positive serum samples for 2 days. After incubation, levels of apoptosis in sample-treated cells were measured using a caspase 3/7 activity assay. For samples carrying polatuzumab vedotin-specific NAbs, the NAbs blocked either the binding of polatuzumab vedotin to CD79b or the internalization of the polatuzumab vedotin/CD79b complexes, leading to reduced caspase 3/7 activity. A patient sample is determined to be NAb positive when it showed a caspase 3/7 activity lower than the plate-specific assay cut-point.

Samples with responses at or below the assay cut-point (i.e., mean normalization control × established cut-point factor, 0.911) were considered NAb-positive, whereas samples with responses above the cut-point were considered NAb-negative.

The presence of polatuzumab vedotin in the sample may interfere with the detection of NAbs in the assay. This assay was determined to detect 1000 ng/mL or 5000 ng/mL of the surrogate positive source material in the presence of either 14.6 μg/mL or 80.0 μg/mL of polatuzumab vedotin, respectively. The relative assay sensitivity was determined to be 650 ng/mL of the surrogate positive source material.

### Positive controls for screening, confirmatory, domain specificity, and titer assays

The positive controls were generated by hyperimmunizing BALB/c mice with an anti-CD79b monoclonal antibody (mAb) (clone 2F2.20.1) in MPL^®^+TDM adjuvant (Millipore Sigma St. Louis, MO). B cells from lymph nodes were harvested from mice demonstrating antibody activity against anti-CD79b mAb and then fused with mouse myeloma cells (PU-1) as previously described (27). Hybridoma clones demonstrating specific anti-anti-CD79b mAb activity were then subcloned by limiting dilution (single cell/well) and screened against anti-CD79b mAb and other human-IgG1 framework recombinant monoclonal antibodies to check for specificity. Selected clones were cultured in INTEGRA CELLine 1000 bioreactors (INTEGRA Biosciences AG, Zizers, Switzerland). The supernatants were then purified by affinity chromatography (MabSelect SuRe; GE Healthcare, Piscataway, NJ), sterile filtered (0.2 µm) and stored at 2°C to 8°C in phosphate buffered saline (PBS).

### Positive control for NAb assay

The NAb assay positive control source was generated by hyperimmunizing New Zealand White rabbits with polatuzumab vedotin to generate polyclonal antibodies against the complementarity determining region of polatuzumab vedotin (Nguyen et al., manuscript in preparation).

Pooled rabbit sera were purified by affinity chromatography (MabSelect SuRe; GE Healthcare, Piscataway, NJ), sterile-filtered (0.2 µm), and stored at -60°C in PBS. Only antibodies that specifically bind to the CDR of polatuzumab vedotin and MMAE were enriched.

The studies were conducted in accordance with the principles of the Declaration of Helsinki, the International Conference on Harmonization E6 Guidelines, and the principles of Good Clinical Practice. Approval from the institutional review boards and ethics committees was obtained before study start. The studies were sponsored by Genentech, Inc. Patient consent was obtained before enrollment.

### Bioanalytical methods for PK analytes

Plasma levels of antibody-conjugated MMAE and unconjugated MMAE were measured using validated liquid chromatography detected by tandem mass spectrometry with and without immunoaffinity capture, respectively, as described previously (23–25).

Serum levels of total antibody were measured using a validated sandwich enzyme linked immunosorbent assay. Diluted samples and controls (MRD 1/100) were added to microtiter plates (Nunc, ThermoFisher Scientific, Waltham, MA) coated with anti-complementarity determining region antibody against polatuzumab vedotin. After a 2-hour incubation, wells were washed and an anti-framework monoclonal antibody conjugated to HRP was added for detection. TMB was added for color development. The minimum quantifiable concentration was 50 ng/mL.

### PK analysis

Three PK analytes were used to characterize polatuzumab vedotin PK (20, 22). Individual PK parameters for antibody-conjugated MMAE, unconjugated MMAE, and total antibody in ADA-positive patients from the pivotal study GO29365 were obtained using Non-Compartmental Analysis (NCA) based on observed data at Cycle 1 and 4, and assessed whether the values are within the range for the overall patient population of each cohort of GO29365 study.

Next, population PK analysis was performed to assess the impact of ADA on the PK of antibody-conjugated MMAE and unconjugated MMAE, using data from studies DCS4968g, GO27834, GO29044 and GO29365 (N=460). Only 12 (2.6%) of 460 patients of the analysis dataset were ADA positive. Due to this low percentage of ADA-positive patients, this factor was not formally assessed as a model covariate. Instead, the simulated exposures using individual Empirical Bayes Estimates parameters with partial covariate correction method (24) were obtained for all patients and compared between ADA+ vs. ADA- group.

## Results

### Integrated summary of immunogenicity

Recent Health Authority guidelines for biologics recommend the inclusion of an Integrated Summary of Immunogenicity in marketing applications (28, 29). Prior to these guidelines, immunogenicity-related information was distributed throughout the marketing applications in various modules. The Integrated Summary of Immunogenicity provides a central location for data and information related to immunogenicity. Here we present information and data included in the polatuzumab vedotin Integrated Summary of Immunogenicity including immunogenicity risk assessment, bioanalytical assay strategy, immunogenicity sample strategy, clinical immunogenicity data analyses, and conclusions on the risks of immunogenicity.

### Analysis of immunogenic risk factors

The immunogenicity assessment utilized a risk-based strategy to evaluate risk factors that may influence polatuzumab vedotin immunogenicity (**Table 1**). Throughout the clinical program, the immunogenicity risk assessment was updated to reflect the current clinical experience. Factors that influence therapeutic immunogenicity can include physiochemical and structural aspects, dosage, administration (frequency, route, and mode), and patient and disease-related factors.

**Table 1:** Immunogenicity risk-based assessment.

Molecule description:	Antibody targeting CD79b conjugated to the microtubule inhibitor monomethyl auristatin E (MMAE) *via* a protease-cleavable peptide linker, maleimidocaproyl-valine-citrulline-p-aminobenzyloxycarbonyl (mc-vc-PAB)
Nature of target/cellular distribution:	CD79b is a cell surface antigen whose expression is restricted to B cells, with the exception of plasma cells. It is expressed in a majority of the B-cell−derived malignancies, including NHL and chronic lymphocytic leukemia
Mechanism of Action:	Polatuzumab vedotin binds CD79b and is rapidly internalized to enable targeted delivery of unconjugated MMAE. Unconjugated MMAE binds to microtubules within the cell and inhibits microtubule polymerization, cell division, and induces apoptosis
Disease treated:	Oncology
Indication(s):	Non-Hodgkin’s Lymphoma (NHL): relapsed or refractory (R/R) Diffuse Large B Cell Lymphoma (DLBCL), treatment-naive DLBCL, R/R follicular lymphoma
Dosing (Route and Frequency):	Intravenous, every 3 or 4 weeks
Patient immune status:	Potentially suppressed due to rituximab treatment
Impact of drug on immune system:	Potential immunosuppressant – B-Cell target
Presence of Pre-existing Immunoreactivity:	No
Endogenous Counterpart:	No
CMC related risks, including antigenic sites, product related variants, and process related impurities:	None identified
Current Immunogenicity Risk Based Assessment (Low, Medium, High):	Low, due to low incidence of anti-drug antibodies in current clinical studies

CMC, Chemistry, Manufacturing, and Controls.

For ADCs, a relatively new class of therapeutic, the theoretical immunogenicity risk was initially considered higher compared to standard monoclonal antibodies (mAbs) given their hapten-like structure (26, 30). The antibody component of polatuzumab vedotin uses a framework similar to those used in other hybridoma-expressed therapeutic IgG1 antibodies. The drug component of polatuzumab vedotin, mc-vc-PAB-MMAE, was known to have minimal immunogenicity risk from clinical trials of Adcetris™, an ADC medication used to treat R/R Hodgkin lymphoma and systemic anaplastic large cell lymphoma (31). Overall, the immunogenicity risk is low based on the mechanism of action, route of administration, no endogenous counterpart, and no manufacturing related risks identified. However, given the novelty of the polatuzumab vedotin antibody and mc-vc-PAB-MMAE drug combination, the molecule was conservatively classified as medium.

Polatuzumab vedotin immunogenicity was not assessed in animal models as the humanized mAb component does not cross-react with rat or cynomolgus monkey CD79b (6). Therefore, during nonclinical development, ADAs were characterized using a surrogate molecule that binds cynomolgus monkey CD79b. Although ADAs were observed in most of the studies, there was minimal impact on exposure and interpretation of toxicology results (6).

In summary, prior to the availability of clinical data, the overall immunogenicity risk for polatuzumab vedotin was initially categorized as medium.

### Physicochemical and structural aspects

The original murine anti-CD79b antibody was humanized using standard procedures, similar to other therapeutic IgG1 mAbs (32–35), and therefore expected to represent a low risk for immunogenicity. The linker-drug (mc-vc-PAB-MMAE) is of non-human origin; in addition, the conjugation through interchain disulfides may result in subtle structural changes to the antibody that potentially exposes neo-epitopes. Therefore the overall risk to induce an immune response was considered medium.

The clinical trials used two different formulations of polatuzumab vedotin drug product: liquid (4 studies) and lyophilized (3 studies). As is typical for IgG1 antibodies, polatuzumab vedotin contains an N-linked glycosylation site in the Fc region of each of the two heavy chains. All glycans observed are typical of human IgG1 isotype (36). Some changes in the distribution of glycoforms were observed when comparing the liquid drug product and the lyophilized drug product. The magnitude of these differences (in levels of high-mannose and other afucosylated glycans) has been shown to not impact overall polatuzumab vedotin PK through a combination of non-clinical and clinical studies (24). No new glycoforms were introduced as a result of the process differences between the liquid drug product and the lyophilized drug product; therefore, an impact on immunogenicity was considered unlikely.

High-molecular-weight forms, which were predominantly molecular dimers of polatuzumab vedotin, were present at low levels (up to 0.8% in lyophilized drug product) and were well controlled by the conjugation process and storage conditions. The extent of this aggregation, which is further controlled by limiting the storage time of the infusion solution, was not expected to contribute to product immunogenicity.

Other product-related species, such as charge variants, sequence variants, subvisible particles, low-molecular-weight forms, process-related impurities, and host cell proteins, that may contribute to immunogenicity risks were controlled through a combination of testing and process control.

### Route and/or mode of administration concerns

Polatuzumab vedotin liquid drug product was filled into vials and stored as a liquid then administered intravenously and undiluted using a syringe pump. Polatuzumab vedotin lyophilized drug product was reconstituted with sterile water then injected into normal saline for administration to R/R DLBCL patients as an infusion.

A minor amount of aggregation (up to 0.1%) was observed following mixing of the reconstituted drug product with normal saline and storage, which can be controlled by limiting storage time in the infusion bag. The slight amount of product aggregation was not expected to increase the risk of immunogenicity relative to administration using a syringe pump and intravenous route of administration was not expected to impact the risk of immunogenicity.

### Patient and disease-related factors

Immunological tolerance in R/R DLBCL patients was not expected to impact polatuzumab vedotin immunogenicity. Next-generation sequencing revealed relatively low numbers of somatic mutations in lymphomas resulting in scant levels of neo-antigens that may limit host immunosurveillance (37). R/R DLBCL patients treated with polatuzumab vedotin were not expected to have developed ADA to polatuzumab vedotin due to previous immunotherapies. While some R/R DLBCL patients may have previously been administered rituximab and could potentially have developed anti-rituximab antibodies that may theoretically cross-react with the antibody component of polatuzumab vedotin, clinical studies have shown that baseline levels of ADAs to polatuzumab vedotin is low (see below). In addition, targeted NHL patient populations that are typically treated with B-cell depleting antibody therapeutics would potentially have suppressed immune systems, reducing the risk of immunogenicity.

### Summary of immunogenicity risk assessment

The current manufacturing processes and physiochemical and structural properties were not expected to meaningfully impact the risk of immunogenicity. In addition, route of administration and patient and disease-related factors were not expected to negatively impact polatuzumab vedotin immunogenicity. The immunogenicity risk assessment was initially categorized as a medium risk during preclinical development, however, once clinical data were available, the immunogenicity risk was updated to low (**Table 1**).

### Assay strategy

**Figure 1:**
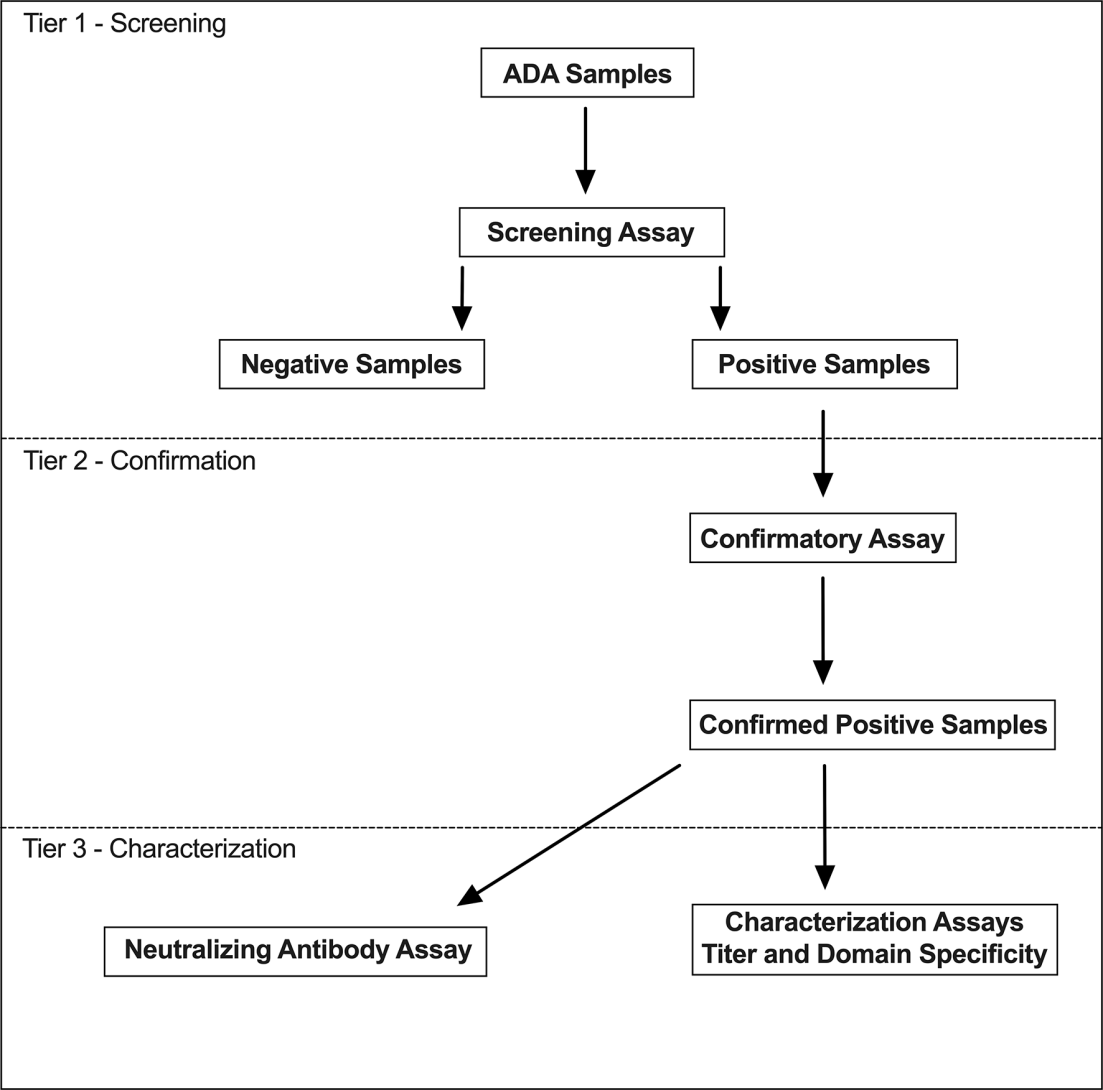
Tiered anti-drug antibody sample analysis strategy.

The immunogenicity assessment strategy was consistent with current health authority guidance for biotherapeutics and industry best practices for ADCs (21, 22, 28, 29). The immunogenicity evaluation used a tiered approach (38), designed to detect and characterize ADA responses to all components of polatuzumab vedotin (**Figure 1**). Validated screening and confirmatory assays were used to assess the immune response in patients treated with polatuzumab vedotin (29). Additional tests were performed on ADA-positive samples to determine the ADA titer and characterize the domain specifically targeted in the response. NAb data were not available at the time of the accelerated submission. As part of a post-marketing commitment, a cell-based apoptosis assay was developed and validated to evaluate the neutralizing activity of ADAs to polatuzumab vedotin.

### Rationale for choice of assays

The rationale for the screening assay was to ensure that ADAs to specific domains of the ADC could be detected with sufficient sensitivity and drug tolerance. The confirmatory assay was designed to assess the specificity of the positive response. The domain specificity was developed to assess, by competitive binding with the antibody component of polatuzumab vedotin, whether the ADA responses were primarily to the antibody portion, the linker-drug regions, or neo-epitopes of the ADC. The rationale for the cell-based NAb assay was to detect the inhibition of tumor cell killing (apoptosis assay) due to the presence of NAb (39).

### Specificity and sensitivity of ADA screening assay

The ADA screening assay minimum required dilution was determined to be 1/50. Acceptable sensitivity and selectivity data observed during development and confirmed during validation indicated that a minimum required dilution of 1/50 was appropriate. A panel of 94 serum samples from polatuzumab vedotin-naïve NHL individuals was run in the ADA screening assay to establish the threshold for ADA-positivity, or screening cut-point. To minimize the potential for false negative results, the screening cut-point was set to yield an untreated positive rate of approximately 5%. The screening assay multiplication (cut-point) factor was determined to be 1.16 times the negative control signal. In neat serum, the relative sensitivities of the polatuzumab vedotin ADA screening assay were estimated to be 60.1 ng/mL using positive control diluted in normal human serum and 1141 ng/mL using an anti-MMAE monoclonal antibody. The screening assay was optimized to tolerate drug interference at the estimated circulating drug level. In the presence of 20 µg/mL of polatuzumab vedotin, two levels of the positive control (90 and 500 ng/mL) tested positive.

The screening assay cut-point factor determined in validation was used to generate a clinical baseline screen positive rate using baseline ADA samples from study GO29365. The in-study baseline screen positive rate was 10.9% (15 out of 137 patients), which falls within the variability for false positive rates for a screening cut-point factor targeting a 5% false positive rate (40). Therefore, following industry practice (40), the assay validation cut-point factor was used and an in-study cut-point factor was not generated.

The screening assay was assessed for rituximab interference and cross-reactivity since this was a co-medication. Rituximab at multiple levels (0, 250, and 600 µg/mL) was added to multiple levels of the positive control (0, 220, 500, and 2000 ng/mL). The samples containing 220-2000 ng/mL positive control and varying levels of rituximab screened positive for ADA, confirming that rituximab did not interfere in the assay. Similarly, the samples containing 0 ng/mL positive control and varying levels of rituximab screened negative for ADA, again confirming rituximab did not appear to interfere nor cross-react in the screening assay.

There have been no published reports of circulating soluble CD79b receptors. Therefore, neither CD79b receptor interference nor cross-interference was investigated.

### Specificity and sensitivity of ADA confirmatory assay

The confirmatory cut-point was determined by analyzing a panel of 94 serum samples from polatuzumab vedotin-naïve NHL individuals in the presence of 30 µg/mL polatuzumab vedotin. The same serum panel used to determine the screening cut-point was used to generate the confirmatory cut-point. The cut-point represents the difference in assay signal between a sample with and without polatuzumab vedotin. To minimize the potential for false negative results, the confirmatory cut-point was set to give an untreated positive rate of approximately 1%. The confirmatory cut-point was a 38% reduction in the ADA signal. Samples that were confirmed positive were then further characterized as described below.

### ADA characterization assay – titer

Samples that were confirmed positive were analyzed in an assay similar to the screening assay to determine titer. Samples were diluted to the minimum required dilution (1/50) and then serially diluted two-fold. Titer values were calculated using the titer offset value (mean negative control signal + 0.0632) and reported as the log_10_ of the sample dilution at which the obtained sample signal would equal the assay cut-point.

### ADA characterization assay – domain specificity

ADCs are multi-domain biotherapeutics. As such, an immune response may be directed against any component of the ADC: antibody, linker, drug, or neo-epitope resulting from the conjugation. Samples that confirmed positive were further characterized in a qualified exploratory characterization assay to assess domain specificity (21, 22, 26, 30). Domain specificity was determined by competitive binding with the antibody portion of polatuzumab vedotin (MCDS4409A). The domain specificity threshold for MCDS4409A positivity was determined by assaying the same panel of 94 therapeutic-naïve individual samples used in the determination of the screening assay multiplication factor. The domain specificity threshold was set to give an untreated positive rate of approximately 1%. The threshold, which represents the difference in assay signal between a sample with and without MCDS4409A, was determined to be 19%. For samples that confirm positive (above the >19% threshold), the antibody response was considered to be primarily directed against the antibody domain of polatuzumab vedotin. For samples that confirm negative, the antibody response was considered to be primarily directed against epitopes unique to polatuzumab vedotin (i.e., towards unconjugated MMAE, linker, or ADC neo-epitopes).

### ADA characterization assay – neutralizing antibodies

ADA-positive samples from confirmed ADA-positive patients were further assessed for NAb activity. A cell based NAb assay was developed and validated.

### Immunogenicity results

The immunogenicity of polatuzumab vedotin was assessed in 7 clinical trials. ADAs against rituximab were not monitored in polatuzumab vedotin studies as the immunogenicity of rituximab in patients with NHL has historically been low (41, 42).

#### Summary of GO29365 immunogenicity results

For study GO29365, the baseline prevalence of ADAs was 3.7% (5/134, **Table 2**). Post-baseline, ADA incidence was 6.0% (8/134). All 8 patients had treatment-induced ADAs (i.e., ADA negative at baseline or missing a baseline sample for ADA analysis and at least one positive post-baseline ADA result). Out of the 8 patients with treatment-induced ADA, 6 patients had a transient response and 2 patients had persistent responses (i.e., ADA positive result detected at the last post-baseline sampling time point or at ≥ 2 time points during treatment where the first and last ADA positive samples are separated by a period ≥ 16 weeks). The 5 patients who tested positive for ADA at baseline were treatment unaffected (ADA response was similar to, or lower than, that at baseline).

**Table 2:** Incidence of ADAs to polatuzumab vedotin in study GO29365.

Disease histology	DLBCL (n=73)				FL (n=71)				All
Study Phase	Ib	II	Ib	II	Ib	II	Ib	II	Ib/II
Cohort/Arm	1a	C	1b	F	1a	A	1b	E	
Sample size (n)	6	40	6	21	6	39	6	20	144
Baseline Prevalence of ADAs									
Baseline evaluable patients	6	36	6	18	6	37	6	19	134
Patients positive for ADA	2 (33.3%)	0 (0.0%)	0 (0.0%)	0 (0.0%)	3 (50.0%)	0 (0.0%)	0 (0.0%)	0 (0.0%)	5 (3.7%)
Patients negative for ADA	4	36	6	18	3	37	6	19	129
Post-Baseline Incidence of ADAs									
Post-baseline evaluable patients	6	35	6	18	6	38	6	19	134
Patients positive for ADA	2 (33.3%)	1 (2.9%)	0 (0.0%)	1 (5.6%)	0 (0.0%)	3 (7.9%)	0 (0.0%)	1 (5.3%)	8 (6.0%)
Treatment-induced ADA	2	1	0	1	0	3	0	1	8
Treatment-enhanced ADA	0	0	0	0	0	0	0	0	0
Patients negative for ADA	4	34	6	17	6	35	6	18	126
Treatment unaffected	2	0	0	0	3	0	0	0	5

ADA, anti-drug antibody; DLBCL, diffuse large B-cell lymphoma; FL, follicular lymphoma.

ADA titers ranged from <1.7 to 2.71 (**Table 3**). Domain specificity indicated that the antibody responses for patients with treatment-induced ADA were directed primarily against the antibody for 2 patients and against the linker, drug, or neo-epitopes for 5 patients. None of the ADA-positive samples demonstrated neutralizing activity. One patient had insufficient sample volume to characterize the immune response. The onset of ADA response varied from as early as after the first dose of polatuzumab vedotin to treatment completion/early termination.

**Table 3:** Characterization of ADA positive samples in study GO29365.

Cohort/Arm	DLBCL			FL				DLBCL	FL	DLBCL
	1a	1a	1a	A	A	A	A	C	E	F
Visit	Pre-dose C2 D1	Pre-dose C2 D1	Pre-dose C4 D1	TC/EW	Pre-dose C2 D1	Pre-dose C4 D1	TC/EW	FUM6	TC/EW	Pre-dose C2 D1
Patient ID	A	B	B	E	F	F	G	C	H	D
ADA Titer	2.26	2.05	2.15	NC	<1.7	<1.7	<1.7	2.42	<1.7	2.71
Pola Total Antibody (µg/mL)	2.34	3.65	6.67	NC	<0.05	3.8	NA	<0.05	7.61	3.09
Pola Antibody Immunodepletion	+	+	+	NC	–	–	–	–	–	–
Duration of ADA Response	Persistent	Transient		Transient	Transient		Transient	Transient	Persistent	Transient
NAb Activity	Negative	Negative	Negative	NC	Negative	Negative	Negative	Negative	Negative	Negative

NAb, neutralizing antibody; ADA, anti-drug antibody; C, cycle; D, day; DLBCL, diffuse large B-cell lymphoma; FL, follicular lymphoma; FUM6, 6 month follow up; NA, not available; NC, not calculable-insufficient sample; pola, polatuzumab vedotin; TC/EW, treatment completion/early withdrawal.

Immunodepletion, competitive binding with the antibody portion of polatuzumab vedotin.

+=positive for immunodepletion; immune response directed mainly at antibody portion.

– =negative for immunodepletion; immune response directed mainly to linker, drug, or neo-epitopes.

Persistent= ADA positive result detected (a) at the last post−baseline sampling time point, OR (b) at 2 or more time points during treatment where the first and last ADA positive samples are separated by a period >= 16 weeks, irrespective of any negative samples in between.

Transient= ADA positive result detected (a) at only one post−baseline sampling time point (excluding last time point) OR (b) at 2 or more time points during treatment where the first and last ADA positive samples are separated by a period of < 16 weeks, irrespective of any negative samples in between.

Polatuzumab vedotin total antibody concentrations corresponding to each ADA sample were determined to understand the potential for drug interference in the ADA results. Out of a total of 531 ADA samples that had measurable polatuzumab vedotin total antibody levels, all samples had levels less than 20 μg/mL, the drug tolerance of the ADA assay. Polatuzumab vedotin total antibody concentrations ranged from <0.05 μg/mL to 10.7 μg/mL with a median concentration of 1.77 μg/mL (data not shown).

The emergence of ADAs to polatuzumab vedotin did not appear to impact efficacy of ongoing long-term responses despite development of ADAs (results not shown). In this study, a total of 8 patients (4 DLBCL, 4 Follicular Lymphoma [FL]) developed ADAs. Of the 4 DLBCL patients, 3 were treated with polatuzumab vedotin with bendamustine plus rituximab (pola+BR) and 1 with polatuzumab vedotin with bendamustine plus obinutuzumab (pola+BG). In the first case, a pola+BR patient (Patient A) developed ADAs during Cycle 2 and was assessed with progressive disease during the Cycle 3 Day 15 interim assessment. The other two pola+BR patients responded to treatment (Patients B and C). Patient B had ADAs detected during Cycle 2, completed all 6 cycles, and has an ongoing duration of response (DOR) of 38.2 months. Patient C had ADAs detected during Follow-up Month 6 visit. This patient completed 5 cycles (discontinued study treatment due to grade 1 muscle loss) and has an ongoing DOR of 21 months. The final DLBCL patient (Patient D, pola+BG) had ADAs detected during Cycle 2, completed 6 cycles, and has an ongoing DOR of 21.1 months.

Four FL patients developed ADAs against polatuzumab vedotin: 3 patients were treated with pola+BR (Patients E, F, and G) and 1 patient with pola+BG (Patient H). One pola+BR patient (Patient F) had ADAs detected during Cycle 2, had a partial response at Cycle 3 Day 15 interim assessment, and progressive disease at primary response assessment. The other 3 patients had ADAs detected at later time points and after completing 6 cycles: 2 at the treatment completion visit (30 days +/- 5 days after the last dose of study treatment) (Patients E and H) and one (Patient G) at the treatment completion visit. All three of these patients (Patients E, G, H) have ongoing responses (durations of 16.0, 21.0, 15.5 months, respectively) at time of analysis.

Safety results for study GO29365 were previously reported (43). Based on available data, there was no identifiable relationship between ADA positivity and reported adverse events.

#### Summary of GO29365 and supportive studies immunogenicity results

Aggregate immunogenicity data for GO29365 and 6 supportive studies for all polatuzumab vedotin treatment groups are shown in **Table 4**. For all patients treated with polatuzumab vedotin, the baseline prevalence of ADAs was 2.4% (13/545). Post baseline, ADAs were detected in 14 of 536 (2.6%) ADA evaluable patients treated with polatuzumab vedotin. Out of the 14 patients positive for ADA, 13 patients had treatment-induced ADA and 1 was treatment-enhanced (positive ADA result at baseline with one or more post-baseline results with titers that are at least 0.60 greater than the baseline titer result). Out of the 13 patients with treatment-induced ADA, 8 patients had a transient response and 5 patients had persistent responses. Out of the 13 patients who tested positive for ADA at baseline, 12 were treatment unaffected. Domain specificity indicated that the immune responses were directed primarily against the antibody for 4 patients and against the linker, drug, or neo-epitopes for 9 patients. One patient had insufficient sample volume to characterize the immune response.

**Table 4:** Incidence of polatuzumab vedotin ADAs in studies DCS4968g, GO27834, GO29044, GO29365, BO29561, GO29833, and GO29834.

Parameter	Clinical Study							All Study Patients (N=641)
	DCS4968g (N=95)	GO27834 (N=231)	GO29044 (N=63)	GO29365 (N=144)	BO29561 (N=34)	GO29833 (N=27)	GO29834 (N=47)	
Baseline evaluable patients	91	160	59	134	31	27	43	545
Patients positive for ADA at baseline	5 (5.5%)	1 (0.6%)	0	5 (3.7%)	0	1 (3.7%)	1 (2.3%)	13 (2.4%)
Patients negative for ADA at baseline	86	159	59	129	31	26	42	532
Post-baseline evaluable patients	91	153	63	134	31	26	38	536
Patients positive for ADA	5 (5.5%)	0	0	8 (6.0%)	0	1 (3.8%)	0	14 (2.6%)
Treatment-induced ADA	4	0	0	8	0	1	0	13
Treatment-enhanced ADA	1	0	0	0	0	0	0	1
Patients negative for ADA	86	153	63	126	31	25	38	522
Treatment unaffected	4	1	0	5	0	1	1	11

ADA, anti-drug antibody.

The ADA response from patients administered either liquid drug product or lyophilized drug product was compared. The overall incidence of ADAs for patients administered with either liquid drug product or lyophilized drug product was 2.9% (13/441) and 1.1% (1/95), respectively. Refer to **Tables S1**, **S2** in Supplementary Data for additional information.

Three PK analytes were used to characterize polatuzumab vedotin PK, as described above. For GO29365, the individual PK parameters for antibody-conjugated MMAE, unconjugated MMAE, and total antibody based on NCA for the 8 ADA-positive patients are listed in **Table 5**. The PK parameters for these patients were within the range for the overall patient population of each cohort of GO29365.

**Table 5:** Individual polatuzumab vedotin PK parameters in patients with positive-ADA status in Study GO29365.

acMMAE													
Treatment Arm	Patient No.		C_max_ (ng/mL)	AUC_inf_ (day*ng/mL)		t_1/2λz_ (day)		CL (mL/day/kg)		V_ss_ (mL/kg)	Cycle 4 pre- dose (ng/mL)		Cycle 4 C_max_ (ng/mL)
Ph Ib DLBCL Pola+BR	A		702	2070		4.86		15.4		69.9	–		–
	B		547	2410		7.38		13.8		110	31.5		648
Arm A FL Pola+BR	E		517	–		–		–		–	9.74		547
	F		422	–		–		–		–	16.8		670
	G		721	–		–		–		–	22.6		–
Arm C DLBCL Pola+BR	C		823	–		–		–		–	6.74		741
Arm E FL Pola+BG	H		940	–		–		–		–	14.0		1080
Arm F DLBCL Pola+BG	D		599	–		–		–		–	25.5		759
Overall Range^a^			402 - 904	1270 - 3900									
Total Antibody													
**Treatment Arm**	**Patient No.**		**C_max_** **(µg/mL)**	**AUC_inf_** (day*µg/mL)		**t_1/2λz_** **(day)**		**CL** **(mL/day/kg)**		**V_ss_** **(mL/kg)**	**Cycle 4 pre-** **dose (µg/mL)**		**Cycle 4** **C_max_** **(µg/mL)**
Ph Ib DLBCL Pola+BR	A		45.2	213		7.23		8.42		68.3	–		–
	B		32.2	259		8.65		7.20		82.0	6.67		41.5
Arm A FL Pola+BR	E		36.2	–		–		–		–	3.40		36.3
	F		29.3	–		–		–		–	3.82		38.7
	G		31.4	–		–		–		–	8.26		–
Arm C DLBCL Pola+BR	C		42.6	–		–		–		–	3.52		41.2
Arm E FL Pola+BG	H		35.5	–		–		–		–	4.57		48.3
Arm F DLBCL Pola+BG	D		28.3	–		–		–		–	5.00		36.1
Overall Range^a^			20.6 – 50.7	116 - 379									
Unconjugated MMAE													
**Treatment Arm**		**Patient No.**			**C_max_** **(ng/mL)**		**Tmax** **(day)**		**AUC_last_** (day*ng/mL)			**Cycle 4 pre- dose** **(ng/mL)**	
Ph Ib DLBCL Pola+BR		A			1.10		5.15		9.96			–	
		B			3.81		4.86		33.2			0.154	
Arm A FL Pola+BR		E			–		–		–			0.0364	
		F			–		–		–			0.119	
		G			–		–		–			0.227	
Arm C DLBCL Pola+BR		C			–		–		–			0.0483	
Arm E FL Pola+BG		H			–		–		–			0.0374	
Arm F DLBCL Pola+BG		D			–		–		–			0.145	
Overall Range^b^					0.365 – 11.2		3.66 – 37.8						

ac, antibody conjugated; ADA, anti-drug antibody; AUC_inf_, area under the concentration-time curve extrapolated to infinity; AUC_last_, area under the concentration-time curve from time zero to time of last measurable concentration; BG, bendamustine plus obinutuzumab; BR, bendamustine plus rituximab; CL, clearance; C_max_, maximum concentration; DLBCL, diffuse large B-cell lymphoma; FL, follicular lymphoma; MMAE, monomethyl auristatin E; PK, pharmacokinetics; pola, polatuzumab vedotin; t_1/2λz_, the first-order terminal half-life; T_max_, time to maximum concentration; V_ss_, steady-state volume of distribution.

a =C_max_ and AUC_inf_ of Cohorts 1a and 1b following first dose.

b =C_max_ and AUC_last_ of Cohorts 1a and 1b following first dose.

C_max_ refers to observations obtained 30-min post-infusion for acMMAE and total antibody. The C_max_ of unconjugated MMAE by NCA may not reflect the true C_max_ due to sparse sampling.

Based on population PK analysis for the PK of ADA-positive patients, the antibody-conjugated MMAE exposures were similar to the ADA-negative patients (<10% difference). The unconjugated MMAE exposures were numerically higher in ADA-positive patients (10% for AUC, 24% for C_max_), but the difference was not statistically significant as indicated by 90% confidence interval of Geometric Mean Ratio containing 1. The magnitude of difference was much smaller compared to CV% of unconjugated MMAE of 44-58%. ADA-positive status does not appear to have a statistically significant or clinically meaningful impact on the PK of antibody-conjugated MMAE and unconjugated MMAE (**Table 6**).

**Table 6:** Comparison of covariate-corrected exposures in ADA-negative patients and ADA-positive.

Exposure	ADA-negative (n=410)		ADA-positive (n=12)	
	GM (CV%)	GMR (90% CI)	GM (CV%)	GMR (90% CI)
acMMAE AUC (ng*day/mL)	2920 (21%)	NA	2710 (29%)	0.928 (0.798-1.08)
acMMAE C_max_ (ng/mL)	732 (15%)	NA	786 (14%)	1.07 (1-1.15)
Unconjugated MMAE AUC (ng*day/mL)	21 (49%)	NA	23.1 (46%)	1.1 (0.863-1.4)
Unconjugated MMAE C_max_ (ng/mL)	1.94 (44%)	NA	2.42 (58%)	1.24 (0.918-1.68)

ac, antibody conjugated; ADA, anti-drug antibody; AUC, area under the concentration-time curve; C_max_, maximum concentration; CV, coefficient of variation; CI, confidence interval; GM, geometric mean; GMR, geometric mean ratio; MMAE, monomethyl auristatin E; NA, not available. Individual exposures were computed following 1.8 mg/kg Q3W dosing for 6 cycles using partial covariate correction procedure.

## Discussion

Theoretically, the potential to elicit an immune response against multi-domain biotherapeutics such as ADCs was originally thought to be greater than for therapeutic mAbs (26). However, Carrasco-Triguero et el. presented the ADA incidence of eight vc-MMAE ADCs across 11 oncology clinical trials. The ADA incidence ranged between 0 and 35.8%, which was within the range previously reported for mAb therapies (26). It must be noted that comparing ADA incidence across products comes with multiple caveats due to patient immune status, therapeutic target, antibody assay format, sensitivity, and drug tolerance. While polatuzumab vedotin was not included in this previous analysis, the linker-drug and conjugation chemistry for these eight ADCs was the same as for polatuzumab vedotin. For seven of the ADCs for which domain specificity was reported, the immune response was predominantly directed to the antibody portion of the ADC (86% - 100% of ADA-positive patients). In contrast, for polatuzumab vedotin studies, 4 of 13 (31%) evaluable patients had antibody responses directed primarily against the antibody domain. Given the variable and generally low immunogenicity observed for ADCs, the hapten-like structure does not appear to increase the overall immunogenic potential.

For Study GO29365, the ADA incidence was low (6.0%). The antibody titers among the 8 ADA-positive patients were also relatively low (<3.0). For ADA-positive samples, the immune response was characterized by assessing various parameters (**Table 3**). There appeared to be no correlation between the onset of ADA, ADA titer, domain specificity, duration of the ADA response, or disease histology. Due to the low number of patients with antibodies against polatuzumab vedotin, no conclusions could be made on the impact of immunogenicity of ongoing efficacy, safety, or exposure.

Polatuzumab vedotin demonstrates a low ADA incidence of 2.6% (14/536) across seven clinical trials. For polatuzumab vedotin, the low ADA incidence is reasonable as polatuzumab vedotin targets dividing B cells and induces apoptosis. In addition to receiving polatuzumab vedotin in combination with rituximab, also a B-cell targeted immunotherapy, the majority of patients previously received first- or second-line rituximab-based therapy, further decreasing the risk of an immune response.

The other ADC presented in the Carrasco-Triguero analysis, ADC A, that was indicated for NHL had a low ADA incidence of 0.7% (1/142). A listing of 7 non-ADC immunotherapeutics approved for the treatment of NHL or chronic lymphocytic leukemia also shows low incidence of ADA (**Table 7**). Two had no immunogenic response (Arzerra (47), Monjuvi (49)) and 4 had ADA incidences less than 5% (Campath (44), Gazyva (46), Rituxan (41), Rituxan Hycela (48), Zevalin (45)). All therapies target immune cells in patients with B-cell lymphoma, which likely reduced the risk of an immune response.

**Table 7:** ADA incidence for non-ADC immunotherapeutics approved for the treatment of NHL or CLL.

Drug	Trade Name	Indication	ADA Incidence^a^
Alemtuzumab	Campath (44)	CLL	1.9% (4/211)
Ibritumomab tiuxetan	Zevalin (45)	Follicular lymphoma	1.3% (6/446)
Obinutuzumab	Gazyva (46)	Follicular lymphoma, CLL	CLL:7% (18/271), GADOLIN: 0%, GALLIUM 0.2% (1/564)
Ofatumumab	Arzerra (47)	CLL	0%
Rituximab	Rituxan (41)	NHL, CLL, rheumatoid arthritis	1.1% (4/356)
Rituximab hyaluronidase	Rituxan Hycela (48)	Follicular lymphoma, diffuse large B-cell lymphoma, CLL	2.00%
Tafasitamab	Monjuvi (49)	Diffuse large B-cell lymphoma	0%

ADA, anti-drug antibody; ADC, antibody-drug conjugates; NHL, Non-Hodgkin’s lymphoma; CLL, chronic lymphocytic leukemia.

a ADA incidence as reported from FDA USPI.

Two manufacturing processes were used to supply polatuzumab vedotin clinical trials. A liquid formulation was used to supply early clinical trials and a lyophilized formulation was used for later clinical trials and for commercialization. Manufacturing process changes may potentially influence immunogenicity depending on potential differences in the product quality attributes (50, 51). However, the differences in the levels of various quality attributes between the two polatuzumab vedotin formulations were not expected to substantially impact the immunogenicity. The incidence of ADAs for patients administered liquid drug product and lyophilized drug product was 2.9% and 1.1%, respectively (**Supplementary Data Tables S1**, **S2**, respectively). The available data do not show a clear difference in ADA incidence for the patients who received the liquid drug product and lyophilized drug product.

## Conclusions

The overall risk of generating an immune response in patients treated with polatuzumab vedotin is considered low based on a variety of considerations, including a comprehensive immunogenicity risk assessment and immunogenicity data from 536 treated patients. Multiple clinical trials demonstrated a low incidence of ADA to polatuzumab vedotin. The low incidence of ADAs is reasonable since the mechanism of action of polatuzumab vedotin is to target and kill B cells, which impacts their ability to develop into plasma cells secreting ADAs. Furthermore, patients are co-medicated with rituximab, which also targets B cells. Therefore, immunogenicity is not expected to impact the benefit/risk profile of polatuzumab vedotin.

## Data availability statement

The original contributions presented in the study are included in the article/**Supplementary Material**. Further inquiries can be directed to the corresponding author.

## Author contributions

RD, VN, and SK conceptualized and implemented the assay strategy. RD, DL, GK, GM, and SK conceptualized the data analyses. RD, RB, DL, TL, GK, GM undertook data analysis and interpretation. RD, DL, and TL collected and assembled the ADA and PK data. RD, RB, DL, TL, and SK wrote the manuscript. All authors critically reviewed and edited draft versions of the paper and approved the final version.
