# Supplementary material for: Integrated summary of immunogenicity of polatuzumab vedotin in patients with relapsed or refractory B-cell non-Hodgkin’s lymphoma
Source: Front Immunol. 2023 Mar 29;14:1119510. doi: 10.3389/fimmu.2023.1119510 (PMC10090561; doi:10.3389/fimmu.2023.1119510)
Supplement: Supplementary file 1 [file Table_1.pdf]

Supplementary Material

Supplementary Data Table S1. Incidence of polatuzumab vedotin ADAs in patients treated with liquid drug product (Studies DCS4968g, GO27834, GO29044, and GO29365)

	**DCS4968g (N=95)**	**GO27834 (N=231)**	**GO29044 (N=63)**	**GO29365 (N=144)**	**All Study Patients (N=533)**
**Baseline Prevalence of ADAs**					
Baseline evaluable patients	91	160	59	134	444
Patients with a positive sample at baseline	5 (5.5%)	1 (0.6%)	0	5 (3.7%)	11(2.5%)
Patients with no positive samples at baseline	86	159	59	129	433
**Post−Baseline Incidence of ADAs**					
Post−baseline evaluable patients	91	153	63	134	441
Patients positive for ADA	5 (5.5%)	0	0	8 (6.0%)	13 (2.9%)
Treatment−induced ADA	4	0	0	8	12
Treatment−enhanced ADA	1	0	0	0	1
Patients negative for ADA	86	153	63	126	428
Treatment unaffected	4	1	0	5	10

ADA = anti−drug antibodies.
Baseline evaluable patient = a patient with an ADA assay result from a baseline sample.
Post−baseline evaluable patient = a patient with an ADA assay result from at least one post−baseline sample.
Number of patients positive for ADA = the number (and percentage) of post−baseline evaluable patients determined to have treatment−induced ADA or treatment−enhanced ADA during the study period.
Treatment−induced ADA = a patient with negative or missing baseline ADA result(s) and at least one positive post−baseline ADA result.
Treatment−enhanced ADA = a patient with positive ADA result at baseline who has one or more post−baseline titer results that are at least 0.60 t.u. greater than the baseline titer result.
Number of patients negative for ADA = number of post−baseline evaluable patients with negative or missing baseline ADA result(s) and all negative post−baseline results, or a patient who is treatment unaffected.
Treatment unaffected = A post−baseline evaluable patient with a positive ADA result at baseline and (a) where all post−baseline titer results are less than 0.60 t.u. greater than the baseline titer result, OR (b) where all post−baseline results are negative or missing.
For any positive sample with titer result less than the minimum reportable titer or any positive sample where a titer cannot be obtained, titer value is imputed as equal to the minimum reportable titer.

Supplementary Data Table S2. Incidence of polatuzumab vedotin ADAs in patients treated with lyophilized drug product (Studies BO29561, GO29833, and GO29834)

	BO29561 (N=34)	GO29833 (N=27)	GO29834 (N=47)	All Study Patients (N=108)
**Baseline Prevalence of ADAs**				
Baseline evaluable patients	31	27	43	101
Patients with a positive sample at baseline	0	1 (3.7%)	1 (2.3%)	2 (2.0%)
Patients with no positive samples at baseline	31	26	42	99
**Post−Baseline Incidence of ADAs**				
Post−baseline evaluable patients	31	26	38	95
Patients positive for ADA	0	1 (3.8%)	0	1 (1.1%)
Treatment−induced ADA	0	1	0	1
Treatment−enhanced ADA	0	0	0	0
Patients negative for ADA	31	25	38	94
Treatment unaffected	0	1	0	1

ADA = anti−drug antibodies.
Baseline evaluable patient = a patient with an ADA assay result from a baseline sample.
Post−baseline evaluable patient = a patient with an ADA assay result from at least one post−baseline sample.
Number of patients positive for ADA = the number (and percentage) of post−baseline evaluable patients determined to have treatment−induced ADA or treatment−enhanced ADA during the study period.
Treatment−induced ADA = a patient with negative or missing baseline ADA result(s) and at least one positive post−baseline ADA result.
Treatment−enhanced ADA = a patient with positive ADA result at baseline who has one or more post−baseline titer results that are at least 0.60 t.u. greater than the baseline titer result.
Number of patients negative for ADA = number of post−baseline evaluable patients with negative or missing baseline ADA result(s) and all negative post−baseline results, or a patient who is treatment unaffected.
Treatment unaffected = A post−baseline evaluable patient with a positive ADA result at baseline and (a) where all post−baseline titer results are less than 0.60 t.u. greater than the baseline titer result, OR (b) where all post−baseline results are negative or missing.
For any positive sample with titer result less than the minimum reportable titer or any positive sample where a titer cannot be obtained, titer value is imputed as equal to the minimum reportable titer.
